# Development and feasibility evaluation of an AR-assisted radiotherapy positioning system

**DOI:** 10.3389/fonc.2022.921607

**Published:** 2022-10-04

**Authors:** Gongsen Zhang, Xinchao Liu, Linlin Wang, Jian Zhu, Jinming Yu

**Affiliations:** ^1^ 1Department of Radiology, Shandong Cancer Hospital and Institute, Shandong First Medical University and Shandong Academy of Medical Sciences, Jinan, China; ^2^ Cancer Center, Shandong University, Jinan, China

**Keywords:** radiotherapy positioning, augmented reality, image visualization, HoloLens 2, accuracy

## Abstract

**Purpose:**

The aim of this study is to develop an augmented reality (AR)–assisted radiotherapy positioning system based on HoloLens 2 and to evaluate the feasibility and accuracy of this method in the clinical environment.

**Methods:**

The obtained simulated computed tomography (CT) images of an “ISO cube”, a cube phantom, and an anthropomorphic phantom were reconstructed into three-dimensional models and imported into the HoloLens 2. On the basis of the Vuforia marker attached to the “ISO cube” placed at the isocentric position of the linear accelerator, the correlation between the virtual and real space was established. First, the optimal conditions to minimize the deviation between virtual and real objects were explored under different conditions with a cube phantom. Then, the anthropomorphic phantom–based positioning was tested under the optimal conditions, and the positioning errors were evaluated with cone-beam CT.

**Results:**

Under the normal light intensity, the registration and tracking angles are 0°, the distance is 40 cm, and the deviation reached a minimum of 1.4 ± 0.3 mm. The program would not run without light. The hologram drift caused by the light change, camera occlusion, and head movement were 0.9 ± 0.7 mm, 1.0 ± 0.6 mm, and 1.5 ± 0.9 mm, respectively. The anthropomorphic phantom–based positioning errors were 3.1 ± 1.9 mm, 2.4 ± 2.5 mm, and 4.6 ± 2.8 mm in the X (lateral), Y (vertical), and Z (longitudinal) axes, respectively, and the angle deviation of Rtn was 0.26 ± 0.14°.

**Conclusion:**

The AR-assisted radiotherapy positioning based on HoloLens 2 is a feasible method with certain advantages, such as intuitive visual guidance, radiation-free position verification, and intelligent interaction. Hardware and software upgrades are expected to further improve accuracy and meet clinicalbrendaannmae requirements.

## 1. Introduction

Radiotherapy is one of the primary treatments for cancers, and more than 50% of patients receive radiation therapy during the course of their illness (1, 2). A critical step of radiotherapy is lying the patient in the correct position on the couch of the linear accelerator for the accuracy of radiation dose delivery. Currently, patient positioning based on treatment room lasers and markers on skin or fixation devices is still routine in most radiotherapy departments. Many techniques were developed to improve the patient positioning accuracy, such as cone beam computed tomography (CBCT) and MRI-Linac. Although these techniques have improved patient positioning, there are some disadvantages: First, it makes radiotherapy more complex and expensive. Second, additional radiation dosages were delivered to the patient, which may cause unexpected consequences. Third, the complicated treatment procedures can increase the therapist’s workload, resulting in fatigue, such as fatigue caused by switching attention between printed treatment plans, screens, lasers, and markers. Last but not least, these techniques can only reflect the positioning errors at the time of scanning but cannot provide real-time and non-rigid positioning guidance (3, 4).

Augmented reality (AR) is a promising visualization technology developed on the basis of virtual reality. It allows people to experience a scenario where virtual and real objects coexist (5). In recent years, AR technology has been increasingly used in medicine, such as education and training (6–8) and hologram-guided surgery (9–11). Talbot et al. first utilized the AR technique to guide the positioning of radiotherapy patients (12), which was subsequently explored and improved by Tarutani et al. (13) and Johnson et al. (14). However, there are some limitations for these methods. On the one hand, the assembly based on display devices, cameras, and computing devices increases the complexity of the system, which is not conducive to convenient technical implementation. The user’s AR experience is significantly compromised due to the phantom’s AR contour being displayed on a two-dimensional screen. On the other hand, the virtual-real patient alignment is based on the operator’s human eye judgment, and there is a lack of effective object tracking methods.

Microsoft HoloLens 2 is a portable head-mounted AR device that integrates multiple necessary hardware and multi-functions such as computing, holographic display, and intelligent interaction, which may provide a solution with AR characteristics for patient positioning. In this paper, an AR-assisted patient positioning system based on HoloLens 2 was developed. A three-dimensional (3D) virtual model generated by treatment planning CT was anchored to the treatment position and visualized by the therapist with HoloLens 2. The innovation is that the therapist can adjust the couch under the guidance of this intuitive hologram and virtual coordinate derived from object tracking, until the real model and virtual model were registered. In addition, Vuforia SDK was used for isocenter calibration, virtual and real space coordinate system establishment, and patient tracking (15). The feasibility and accuracy of the system were evaluated in the clinical environment. As far as we know, our system is the first radiotherapy positioning method solely based on a head-mounted AR device, providing 3D object tracking and virtual coordinate indication.

## 2. Methods and materials

### 2.1 System overview

**Figure 1:**
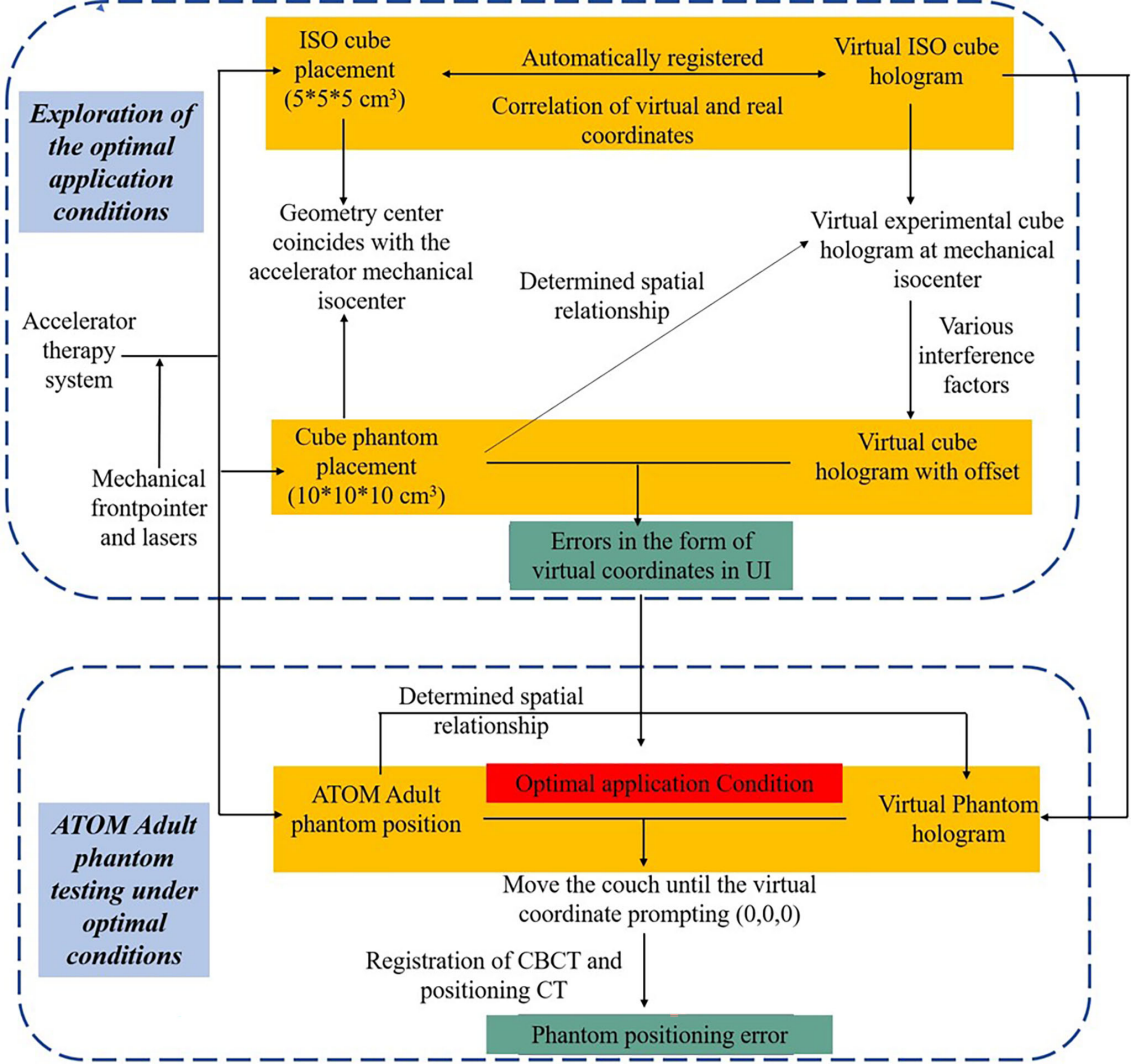
The overall experimental for the proposed AR-assisted positioning system.

The system provides assistance for radiotherapy therapists to perform radiotherapy positioning under an intuitive holographic guidance. In our proposed AR-assisted method, HoloLens 2 was the only required hardware. Moreover, a proprietary SDK, Vuforia, was introduced and worked with the front-facing cameras of HoloLens 2 for automatic registration and real-time tracking, which improved the stability of anchored hologram in physical space to a certain extent and achieved good registration accuracy. In addition to system development and data preparation, we also designed a complete experimental process from optimal conditions exploration to phantom testing in the actual radiotherapy positioning clinical environment, as shown in **Figure 1**.

### 2.2 3D reconstruction and visualization of CT simulation image

**Figure 2:**
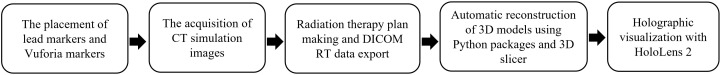
The flow from CT simulation image acquisition to holographic model visualization.

One “ISO cube” (5 × 5 × 5 cm), one cube phantom (10 × 10 × 10 cm), and an anthropomorphic phantom (ATOM Dosimetry verification phantom) were used in this experiment. Lead markers were attached to the surface of phantoms according to the clinical routine. Vuforia markers were also attached to the “ISO cube” and two phantoms. CT simulation images were obtained with SIEMENS SOMATOM Confidence and uploaded to Varian Eclipse treatment planning system. Slice thickness was 3 mm. The treatment plan was formulated on the basis of the simulation CT. To simplify the experimental procedures, the reference points determined by three tiny lead markers were artificially defined as the isocenter. The DICOM RT images were exported to Python package and 3D Slicer programs to automatically reconstruct 3D models. Then, the 3D models and pre-designed user interface (UI) were imported into the AR scene and deployed to HoloLens 2 for holographic visualization (**Figure 2**).

### 2.3 Registration between real and virtual space

**Figure 3:**
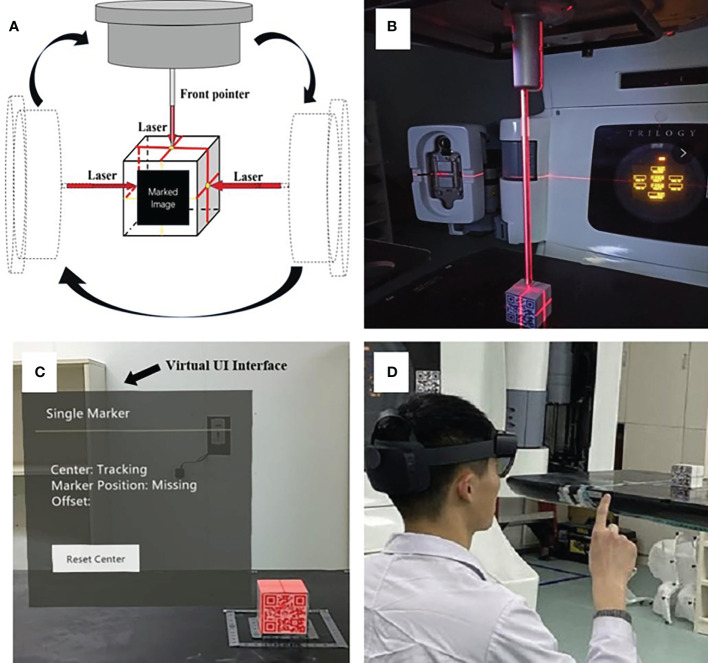
The establishment of the correlation between virtual and real space: precise placement of the “ISO cube” in the isocentric position with the aid of lasers and the mechanical front-pointer **(A, B)**; automatic registration of virtual “ISO cube” and real “ISO cube” based on the HoloLens 2’s detection of Vuforia marker, and the arrow points to the virtual UI interface **(C)**; the gesture interaction to anchor the correlation between virtual and real space **(D)**.

The lasers have been professionally calibrated before the experiment. An “ISO cube” (5 × 5 × 5 cm) used for daily QA (quality assurance) was used as a “registration cube”, and it was placed at an isocentric position so that its geometric center coincided with the mechanical isocenter of the linear accelerator with the aid of room lasers and the mechanical front-pointer (**Figure 3**). After the therapist put on the HoloLens 2 and initiated the test procedure, the front-facing camera of the HoloLens 2 detected the Vuforia marker attached to the “ISO cube”, the virtual cube would automatically be registered to the “ISO cube” (**Figure 3**). The therapist could perform the voice command “fixed” or the gesture interaction (**Figure 3**) to anchor the virtual “ISO cube”, and the correlation between virtual and real space coordinates was established. At the same time, the holographic phantom would be displayed on the HoloLens 2 automatically.

### 2.4 Cube phantom and ATOM phantom test

#### 2.4.1 Cube phantom test

**Figure 4:**
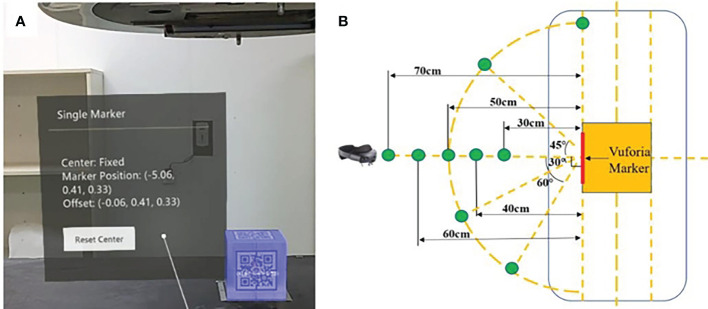
Display of offset between virtual and real geometric centers in virtual UI interface **(A)** and the units of coordinate are in centimeters; the schematic diagram of partial exploratory conditions **(B)**.

Like the procedure for positioning “ISO cube”, a cube phantom (10 × 10 × 10 cm) was placed at the isocenter of the linear accelerator. After the HoloLens 2 detected the Vuforia marker attached to the cube phantom, the position coordinates of the Vuforia marker in the established virtual space coordinate system were displayed in the virtual UI interface. Meanwhile, the coordinates of the geometric center of the cube phantom were also calculated on the basis of the known space relationships between it and Vuforia marker and were displayed. The “Offset” in the virtual UI interface reflected the deviation between the virtual and real geometric center (**Figure 4**). In addition, the spatial deviation was calculated with the following formula, and the results were averaged to determine the overall error:


(1) $$D=\sqrt{X^{2}+Y^{2}+Z^{2}}$$

where *X*, *Y*, and *Z* denote the coordinates of the real geometric center in directions of lateral, vertical, and longitudinal, respectively, of the established virtual space coordinates. The factors that can impact the system’s accuracy were tested, including different angles, different distances, different room light intensities, and with or without camera occlusion and head movement (**Figure 4**). Different angles, including registration angles and tracking angles, refer to the angle between the HoloLens 2’s front-facing camera and the Vuforia marker on the “ISO cube” or the Vuforia marker on the cube phantom, respectively: 0° refers to the direction perpendicular to the Vuforia marker; 30°, 45°, 60°, and 90° are the included angles between the HoloLens 2’s front camera and the vertical direction, respectively, in the horizontal plane. The distances between the front camera of HoloLens 2 and the Vuforia marker are 30, 40, 50, 60, and 70 cm. The light intensity in the treatment room for daily use is divided into normal light intensity (405.0 Lux) and low light intensity (230.0 Lux). For camera occlusion, the therapist artificially covers the front camera of HoloLens 2 for a few seconds when the holographic phantom was displayed on the HoloLens 2. The spatial drift of hologram caused by light changes, camera occlusion, and head movement can be evaluated with the variations of the above formula:


(2) $$D=\sqrt{{\left(X_{2}-X_{1}\right)}^{2}+{\left(Y_{2}-Y_{1}\right)}^{2}+{\left(Z_{2}-Z_{1}\right)}^{2}}$$

where *X*_1_, *Y*_1_, and *Z*_1_ and *X*_2_, *Y*_2_, and *Z*_2_, denote the coordinates of the real geometric center before and after light intensity, camera occlusion, and head movement changes, respectively.

#### 2.4.2 Anthropomorphic phantom test

**Figure 5:**
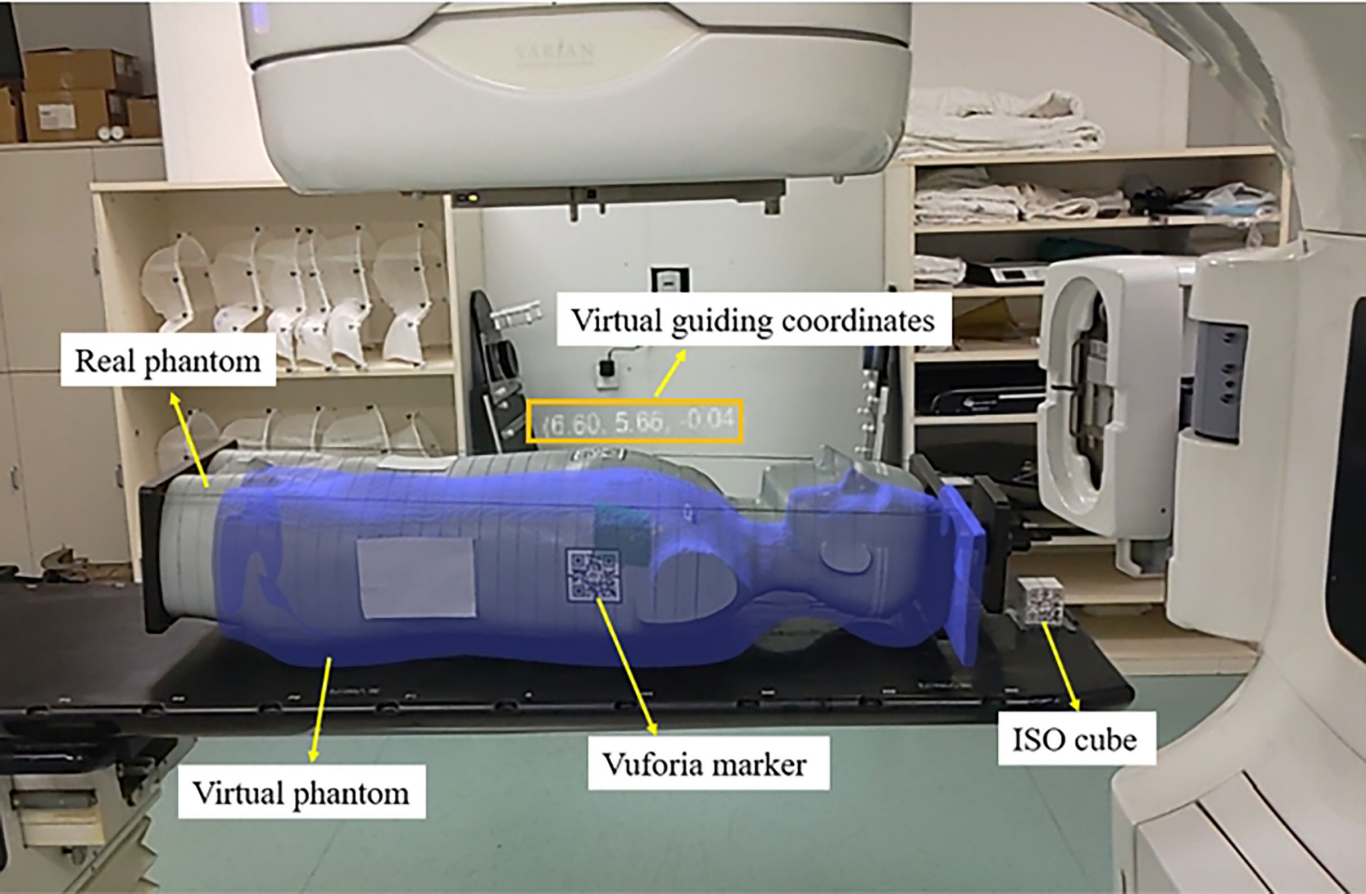
The AR scene of the therapist’s perspective shows a virtual anthropomorphic phantom in the treatment position.

**Figure 6:**
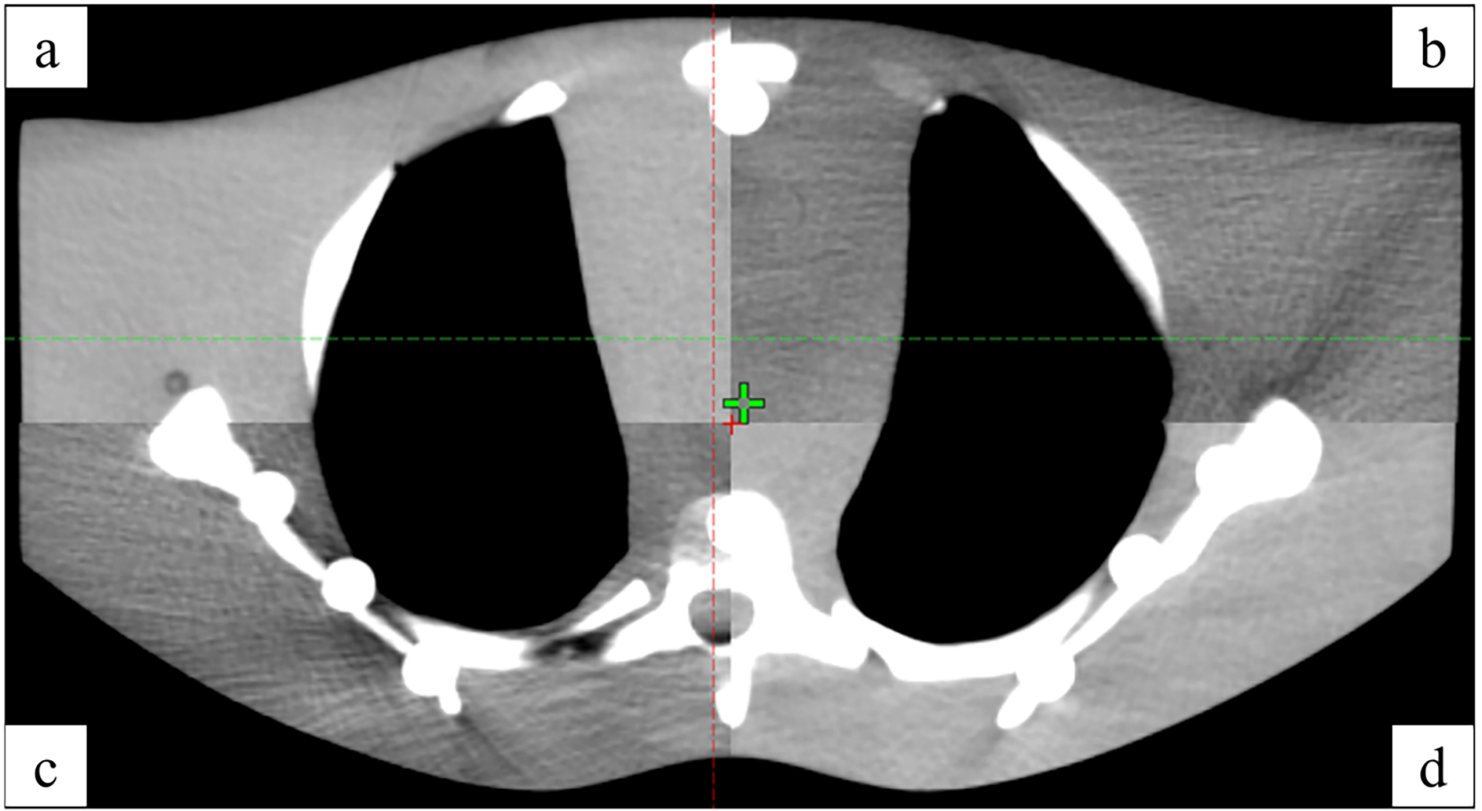
The registration between CBCT **(A, D)** and simulated positioning CT **(B, C)**.

An anthropomorphic phantom was used to test the HoloLens 2–based patient positioning system in the clinical environment (**Figure 5**). After the registration between real and virtual space was established under the optimal exploratory conditions and the holographic anthropomorphic phantom (blue model) in the treatment position was displayed, the therapist moved the treatment couch with the rough guidance of holographic phantom and the fine instructions of virtual coordinates until the virtual coordinates prompting 0,0,0. Then, CBCT was performed to evaluate the positioning errors (**Figure 6**).

#### 2.4.3 Statistical analysis

The experimental data were presented as mean ± SD and were analyzed by paired Student’s t-tests. P<0.05 was considered to represent a statistically significant difference.

## 3. Results

### 3.1 Exploration of optimal conditions

The factors affecting accuracy and stability were confounding, and a method of controlling variates was adopted in the measurement process. The registration angle, tracking angle, and tracking distance affect the recognition and tracking of the Vuforia marker, which has further influence on the coordinate construction and the spatial position accuracy of the holographic model in the AR scenes. For the registration angle and tracking angle, we tested the system performance at 0°, 30°, 45°, and 60°, respectively, with a total of 40 measurements; for the distance, we tested between 30 and 70 cm with a step of 10 cm, and the total number of tests is 50. In addition, the light intensity, camera occlusion, and head movement may affect the accuracy and stability of signal feedback and detection of the depth sensors. The tests for the above three factors were performed 20 times, respectively. The results are summarized in **Tables 1**–**3**. The results of different registration angles were first recorded under the conditions that the tracking angle was consistent with the registration angle, the distance was 50 cm, light intensity was normal, and there was no camera occlusion and head movement. The results showed that there was no significant difference between 0° and 45° (P = 0.84), and significant differences between 0° and other angles (as shown in **Table 1**). Because 0° was easier to control, it was chosen as one of the optimal conditions. Next, the results of different tracking angles were recorded under the registration angle of 0°, and other conditions were the same as above, demonstrating significant differences between 0° and other angles (as shown in **Table 2**). Therefore, the tracking angle was consistent with the registration angle, and both were 0°, which can be regarded as one of the best conditions for further exploration. Then, the results of different tracking distances were recorded under the conditions that the registration angle and the tracking angle were 0°, light intensity was normal, the space deviation was minimized to 1.4 ± 0.3 mm at a distance of 40 cm, and there were significant differences between 40 cm and other distances (as shown in **Table 3**). The results of different light intensity in the treatment room under above optimal conditions were recorded, demonstrating no significant differences between the normal and the low light intensity (P = 0.83). The hologram drift produced by the light changes is 0.7 ± 0.4 mm. However, the program will not work without lights. The hologram drift caused by camera occlusion and head movement were 1.0 ± 0.6 mm and 1.5 ± 0.9 mm, respectively (**Table 4**). On the basis of the results above, the optimal conditions can be summarized as follows: the registration angle and the tracking angle were consistent at 0°, the distance was 40 cm, light intensity was normal, and there was no camera occlusion and head movement.

**Table 1:** Errors at different registration angles.

Angle (degrees)	X (mm)	Y (mm)	Z (mm)	D (mm)	P_1_
0	0.8 ± 0.4	0.9 ± .5	1.2± ± 0.7	1.9 ± 0.5	–
30	2.4 ± 0.6	0.7 ± 0.4	0.3 ± 0.2	2.5 ± 0.6	0.04
45	1.6 ± 0.4	0.4 ± 0.3	0.6 ± 0.3	1.8 ± 0.5	0.84
60	2.3 ± 0.6	0.9 ± 0.6	2.6 ± 0.9	3.7 ± 0.9	<0.01

Statistical significance P_1_: 0 degree vs. other registration angles.

**Table 2:** Errors at different tracking angles.

Angle (degrees)	X (mm)	Y (mm)	Z (mm)	D (mm)	P_2_
0	0.8 ± 0.4	0.9 ± 0.5	1.2 ± 0.7	1.9 ± 0.5	–
30	1.3 ± 1.1	2.0 ± 0.9	1.2 ± 0.9	3.0 ± 1.1	0.01
45	2.0 ± 1.6	2.1 ± 0.7	1.8 ± 1.8	3.9 ± 1.4	<0.01
60	1.9 ± 1.5	2.3 ± 0.8	2.4 ± 1.8	4.3 ± 1.4	<0.01

Statistical significance P_2_: 0 degree vs. other tracking angles.

**Table 3:** Errors at different tracking distances.

Distance (cm)	X (mm)	Y (mm)	Z (mm)	D (mm)	P_3_
30	0.9 ± 0.5	0.9 ± 0.3	1.1 ± 0.5	1.8 ± 0.3	<0.01
40	0.7 ± 0.3	1.0 ± 0.4	0.6 ± 0.4	1.4 ± 0.3	–
50	0.8 ± 0.4	0.9 ± 0.5	1.2 ± 0.7	1.9 ± 0.5	0.02
60	1.4 ± 1.0	1.4 ± 0.9	1.7 ± 0.9	3.2 ± 1.5	<0.01
70	2.0 ± 1.4	1.7 ± 1.0	1.8 ± 0.7	3.5 ± 1.0	<0.01

Statistical significance P_3_: 40 cm vs. other tracking distances.

**Table 4:** The hologram drifts are caused by the light intensity change, camera occlusion, and head movement.

Drift factors	Light intensity change	Camera occlusion	Head movement
Mean (mm)	0.7	1.0	1.5
SD (mm)	0.4	0.6	0.9

### 3.2 Accuracy results of the anthropomorphic phantom

The positioning errors in the X, Y, and Z directions were 3.1 ± 1.9 mm, 3.0 ± 2.8 mm, and 4.6 ± 2.8 mm, respectively. In addition, the angle deviation of Rtn was 0.26 ± 0.14°.

## 4. Discussion

In this study, an innovative AR-assisted radiotherapy positioning system was developed with HoloLens 2 as the only core hardware and provided patient tracking and virtual coordinate indication for radiation therapist, instead of relying on the human eye for virtual-real alignment (12–14, 16). Compared with the related works, this improves the convenience (12, 16), accuracy (17), and practicality of AR guidance systems. The feasibility and accuracy of this method were evaluated in the actual clinical environment. Because of the high-precision requirement of radiotherapy positioning, it is necessary to fully explore the positioning method based on the HoloLens 2 and Vuforia SDK, avoiding the factors that increase the offset between the virtual object and real object. Opposite ideas were adopted in the two stages of the experimental process. We adopted fixing the real object and then watching the virtual coordinates to explore optimal conditions based on the cube phantom. On the contrary, in the anthropomorphic phantom positioning stage, the real object was transferred under the guidance of the virtual coordinates to simulate the actual process. The results from the optimal condition exploration stage show that some factors indeed influence the accuracy of the proposed AR-assisted system, such as the distance between HoloLens 2 and Vuforia marker, as well as the registration and tracking angles, which were related to the size and plane attributes of the Vuforia marker used, respectively. In addition, camera occlusion and head movement caused hologram drift, and the randomness of drift may be brought more significant positioning errors to a certain extent.

Furthermore, the change of indoor light intensity for daily use had no significant effect on the results. Our experiments found that the virtual coordinates were prone to fluctuations, so we obtained relatively stable readings. However, the optimal application conditions obtained were not fully and accurately applied to anthropomorphic phantom–based positioning tests, except for the registration step based on the “ISO cube”. For one thing, the acquisition of real-time coordinates depended on the real-time tracking of the Vuforia marker attached to the anthropomorphic phantom. However, it was difficult for the therapist to maintain the optimal distance, tracking angle, and stationary head. For another, unlike the cube phantom, although we used a relatively flat chest, the surface still has a specific curvature, which led to the pattern distortion of the Vuforia marker and may impact the recognition and tracking process. We also found that the heavier anthropomorphic phantom caused the bed to settle, unlike the lighter cube phantom. At the same time, the virtual phantom remained in its original position, resulting in an inherent positional deviation between the virtual and real phantom that ultimately affected positioning accuracy. The good thing was that the bed settlement could be roughly corrected according to the general outline of the virtual phantom.

The results of the anthropomorphic phantom showed that, although the accuracy is in millimeter scale, it is still not up to the requirements of clinical use at present. However, considering the advantages that this method does bring. First, the virtual 3D model is directly presented in the treatment position so that the patient positioning process is more intuitive and straightforward. Second, the decrease of CBCT frequency reduces the cost of treatment and non-treatment dose for patients. Third, the valuable information is presented in real time in virtual form, which realizes paperless and screenless and solves the problem of attention shift, and optimizes human ergonomics with the adjustment of virtual UI interface (18) in the process of positioning. Fourth, unlike CBCT, which can only collect position information at a particular moment, AR-assisted positioning can continuously monitor and correct the patient’s position. Fifth, in combination with other artificial intelligence technology can realize the automatic identification of patients or fixed appliances to avoid human error. Finally, it helps radiologists remotely guide the patient’s radiotherapy positioning and bring high-quality patient education by presenting previously invisible beams, target areas, and organs at risk.

Therefore, it is necessary for us to continue to explore ways to reduce the positioning error in future work. On the one hand, we will consider two or more HoloLens 2 to collaborate, share, and exchange information and realize the complementation of spatial information. On the other hand, considering the limitation caused by the planar Vuforia marker and inspired by the surface-guided radiation therapy technology, we will try to adopt binocular stereo vision or structured light to obtain the overall surface information of the phantom as the basis for 3D holographic image reconstruction. In the future, we look forward to advancing hardware and software to make automatic registration more accurate and the spatial hologram more stable.

## 5. Conclusion

We developed an AR-assisted positioning system for radiotherapy based on HoloLens 2 and evaluated its feasibility and accuracy. The results showed that the system’s accuracy is in millimeters, which roughly meets clinical requirements and still needs to be further improved. However, considering the advantages, including intuitive visual guidance, radiation-free position verification, and intelligent interaction, the proposed AR method has a promising future.

## Data availability statement

The raw data supporting the conclusions of this article will be made available by the authors, without undue reservation.

## Author contributions

All authors contributed to the study's conception and design. Material preparation, data collection, and analysis were performed by GZ, XL, and LW. All authors read and approved the final manuscript.

## Funding

This work was supported by the National Natural Science Foundation of China (grant numbers 8217102892, 81972863, 81627901, and 82030082) and Natural Science Foundation of Shandong Province (grant numbers ZR2019LZL012).

## Conflict of interest

The authors declare that the research was conducted in the absence of any commercial or financial relationships that could be construed as a potential conflict of interest.

## Publisher’s note

All claims expressed in this article are solely those of the authors and do not necessarily represent those of their affiliated organizations, or those of the publisher, the editors and the reviewers. Any product that may be evaluated in this article, or claim that may be made by its manufacturer, is not guaranteed or endorsed by the publisher.
